# Increasing iterative reconstruction strength at low tube voltage in coronary CT angiography protocols using 3D‐printed and Catphan^®^ 500 phantoms

**DOI:** 10.1002/acm2.12977

**Published:** 2020-07-13

**Authors:** Kamarul A. Abdullah, Mark F. McEntee, Warren M. Reed, Peter L. Kench

**Affiliations:** ^1^ Faculty of Health Sciences Universiti Sultan Zainal Abidin (UniSZA) Kuala Terengganu Malaysia; ^2^ Medical Imaging Optimisation and Perception Group (MIOPeG) Discipline of Medical Imaging Science Faculty of Medicine and Health Sydney School of Health Sciences The University of Sydney Camperdown NSW Australia

**Keywords:** 3D printing, CT, image quality, iterative reconstruction, phantom, radiation dose

## Abstract

**Purpose:**

The purpose of this study was to investigate the effect of increasing iterative reconstruction (IR) algorithm strength at different tube voltages in coronary computed tomography angiography (CCTA) protocols using a three‐dimensional (3D)‐printed and Catphan^®^ 500 phantoms.

**Methods:**

A 3D‐printed cardiac insert and Catphan 500 phantoms were scanned using CCTA protocols at 120 and 100 kVp tube voltages. All CT acquisitions were reconstructed using filtered back projection (FBP) and Adaptive Statistical Iterative Reconstruction (ASIR) algorithm at 40% and 60% strengths. Image quality characteristics such as image noise, signal–noise ratio (SNR), contrast–noise ratio (CNR), high spatial resolution, and low contrast resolution were analyzed.

**Results:**

There was no significant difference (*P *> 0.05) between 120 and 100 kVp measures for image noise for FBP vs ASIR 60% (16.6 ± 3.8 vs 16.7 ± 4.8), SNR of ASIR 40% vs ASIR 60% (27.3 ± 5.4 vs 26.4 ± 4.8), and CNR of FBP vs ASIR 40% (31.3 ± 3.9 vs 30.1 ± 4.3), respectively. Based on the Modulation Transfer Function (MTF) analysis, there was a minimal change of image quality for each tube voltage but increases when higher strengths of ASIR were used. The best measure of low contrast detectability was observed at ASIR 60% at 120 kVp.

**Conclusions:**

Changing the IR strength has yielded different image quality noise characteristics. In this study, the use of 100 kVp and ASIR 60% yielded comparable image quality noise characteristics to the standard CCTA protocols using 120 kVp of ASIR 40%. A combination of 3D‐printed and Catphan^®^ 500 phantoms could be used to perform CT dose optimization protocols.

## 1 INTRODUCTION

Coronary computed tomography angiography (CCTA) has emerged as a powerful imaging tool for diagnosing coronary artery disease (CAD).^1^, ^2^, ^3^ Recently, the number of multislice CT scanners available has increased tremendously. As a result, the number of CCTA scans performed is also likely to increase.^4^ Coronary computed tomography angiography contributes an amount of radiation dose to the patients which carries risks of cancer, especially to the radiosensitive organs.^5^ In a previous multicenter study,^6^ the authors reported the mean effective dose of CCTA could achieve up to 12 mSv among the participating centers. Therefore, an effective strategy to further reduce the dose while maintaining the image quality is highly desirable.

Coronary computed tomography angiography is usually performed with a tube voltage of 120 kVp.^7^, ^8^ Scanning acquisitions at a low tube voltage of 100 kVp has been suggested as an effective way to reduce radiation dose in non‐obese patients while maintaining image quality.^9^, ^10^ The low tube voltage helps to enhance coronary vessels as a result of the increased attenuation of iodinated contrast material.^11^, ^12^ However, this low tube voltage can also deteriorate the image quality by increasing the image noise.^13^

Iterative reconstruction (IR) algorithm can compensate the increment of noise, especially when using the low exposure factors.^14^, ^15^ As a result, dose reduction will be achieved while maintaining image quality. Iterative reconstruction also offers a selection of strength levels resulting in different amounts of noise reduction.^16^, ^17^ However, high IR strengths may result in “blooming” artifacts that typically affect the visualization of small structures.^18^ As a result, the level of the IR algorithm strengths must be carefully considered so as to balance its impact on image quality.

Previous literature had studied and established the use of three‐dimensional (3D)‐printed cardiac insert phantom for evaluating the effect of IR strengths on image quality and dose reduction potential.^19^, ^20^, ^21^ The cardiac insert phantom was constructed using a 3D printing technology. In contrast to other phantoms, such as Catphan^®^ (Phantom Laboratory, Salem, NY) and ACR (American College of Radiology), this phantom can be placed in an anthropomorphic chest phantom. Therefore, this combination provides an appropriate simulation of a patient’s attenuation properties during CCTA along with anatomically relevant cardiac structures for the assessment of image quality.

The purpose of this study was to investigate the effect of changing iterative reconstruction (IR) algorithm strength at different tube voltages in CCTA protocols using a 3D‐printed and Catphan^®^ 500 phantoms.

## 2 MATERIALS AND METHODS

### 2.A. Phantoms

**Fig. 1:**
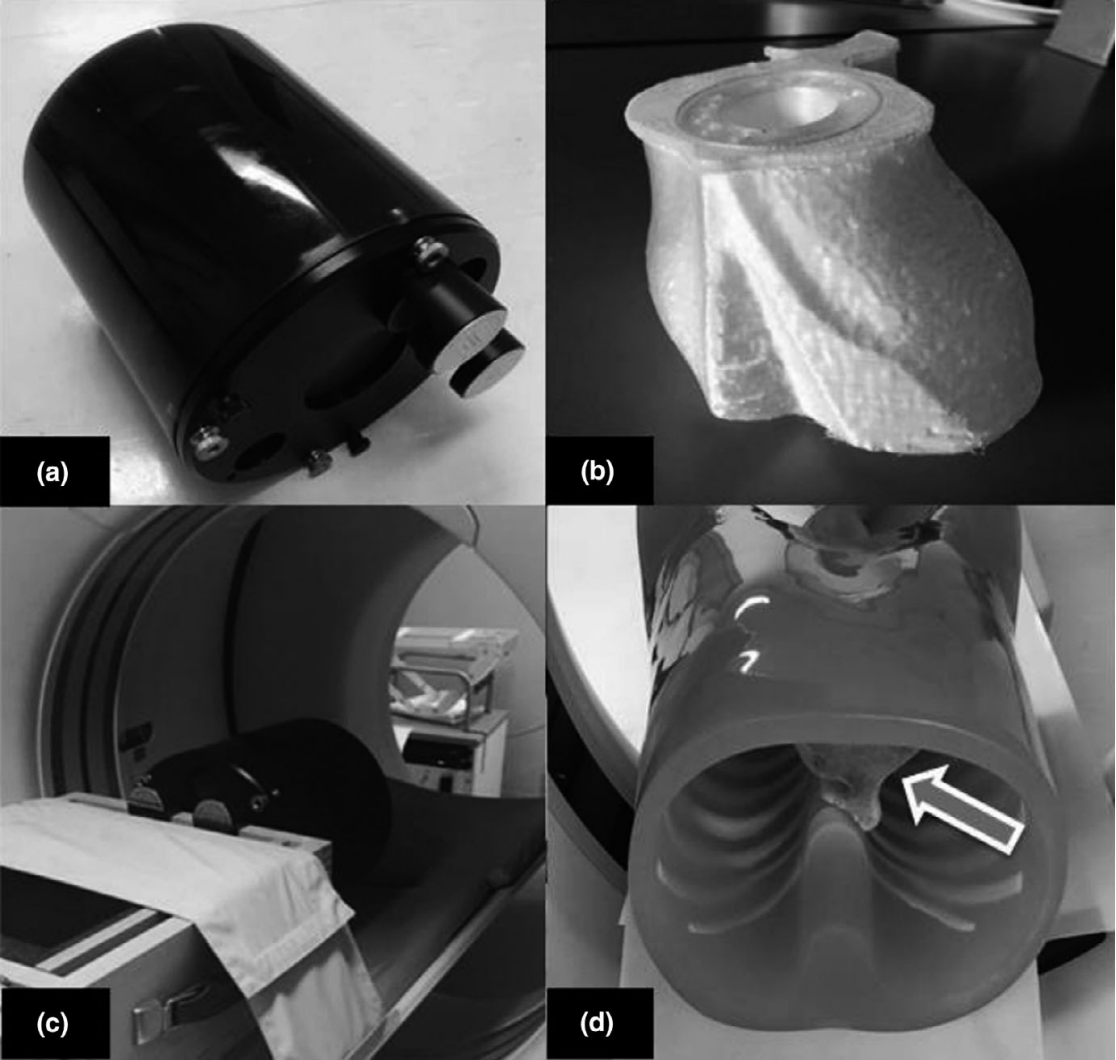
(a) The Catphan^®^ 500 phantom (The Phantom Laboratory, Greenwich, NY, USA); (b) the three‐dimensional (3D)‐printed cardiac insert phantom; (c) the Catphan^®^ 500 phantom was positioned on the scanner table; and (d) the anthropomorphic chest phantom (Lungman N‐01, Kyoto Kagaku, Japan), with the 3D‐printed cardiac insert phantom positioned within (arrow), was placed on the scanner table.

Two phantoms were used in this present study, (a) 3D‐printed cardiac insert phantom, and a (b) Catphan^®^ 500 (The Phantom Laboratory, Greenwich, NY, USA) phantom (see Fig. 1). The 3D‐printed cardiac insert phantom, simulating the contrast‐enhanced region of the ascending aorta and coronary vessels in CCTA, was constructed using 3D printing technology and placed within an anthropomorphic chest phantom (Lungman N‐01, Kyoto Kagaku Co., Ltd., Kyoto, Japan). The development of the 3D‐printed cardiac insert phantom was described in our previous work.^22^ The contrast medium used was Ultravist 370 (Schering Health Care Ltd, Burgess Hill, UK). For the Catphan^®^ 500 phantom, two modules of CTP528 and CTP515 were included. The modules were used to assess the axial spatial resolution and low contrast detectability.

### 2.B. Acquisitions and reconstruction

The 3D‐printed cardiac insert phantom was positioned within the Lungman phantom. The Lungman and the Catphan^®^ 500 phantoms were scanned using 64‐slice SPECT/CT scanner (Discovery 570c, GE Healthcare, Milwaukee, WI, USA). Both phantoms were scanned at 120 and 100 kVp tube voltages with auto mA settings resulting in two CT dose indices volume (CTDI_vol_) of 4.27 and 1.82 mGy, respectively. For the IR algorithm, the adaptive statistical iterative reconstruction (ASIR, GE Healthcare, Milwaukee, WI, USA) was used. Filtered back projection (FBP) and two different IR strengths of ASIR 40% and 60% were investigated. The 120 kVp and ASIR 40% are the current CCTA protocols used in the institution where the phantom was scanned. Table 1 shows the summary of CT parameters and reconstruction settings used in this present study.

**Table 1:** Summary of computed tomography (CT) parameters and reconstruction settings.

Parameters	
Collimation (mm)	0.625
Tube current (mA)	Auto
Tube voltage (kVp) (CTDI_vol_ (mGy))	120 (4.27), 100 (1.82)
Reconstruction settings	FBP, ASIR 40%, ASIR 60%

ASIR, adaptive statistical iterative reconstruction; FBP, filtered back projection.

### 2.C. Image quality assessment

Image quality was calculated using ImageJ software (v1.46r, National Institutes of Health, Bethesda, MD, USA, http://imagej.nih.gov/ij/) for the 3D‐printed cardiac insert phantom, and AutoQA Lite^TM^ program (v3.1.5.7, Iris QA, LLC, Maryland, USA) for the Catphan^®^ 500 phantom.

**Fig. 2:**
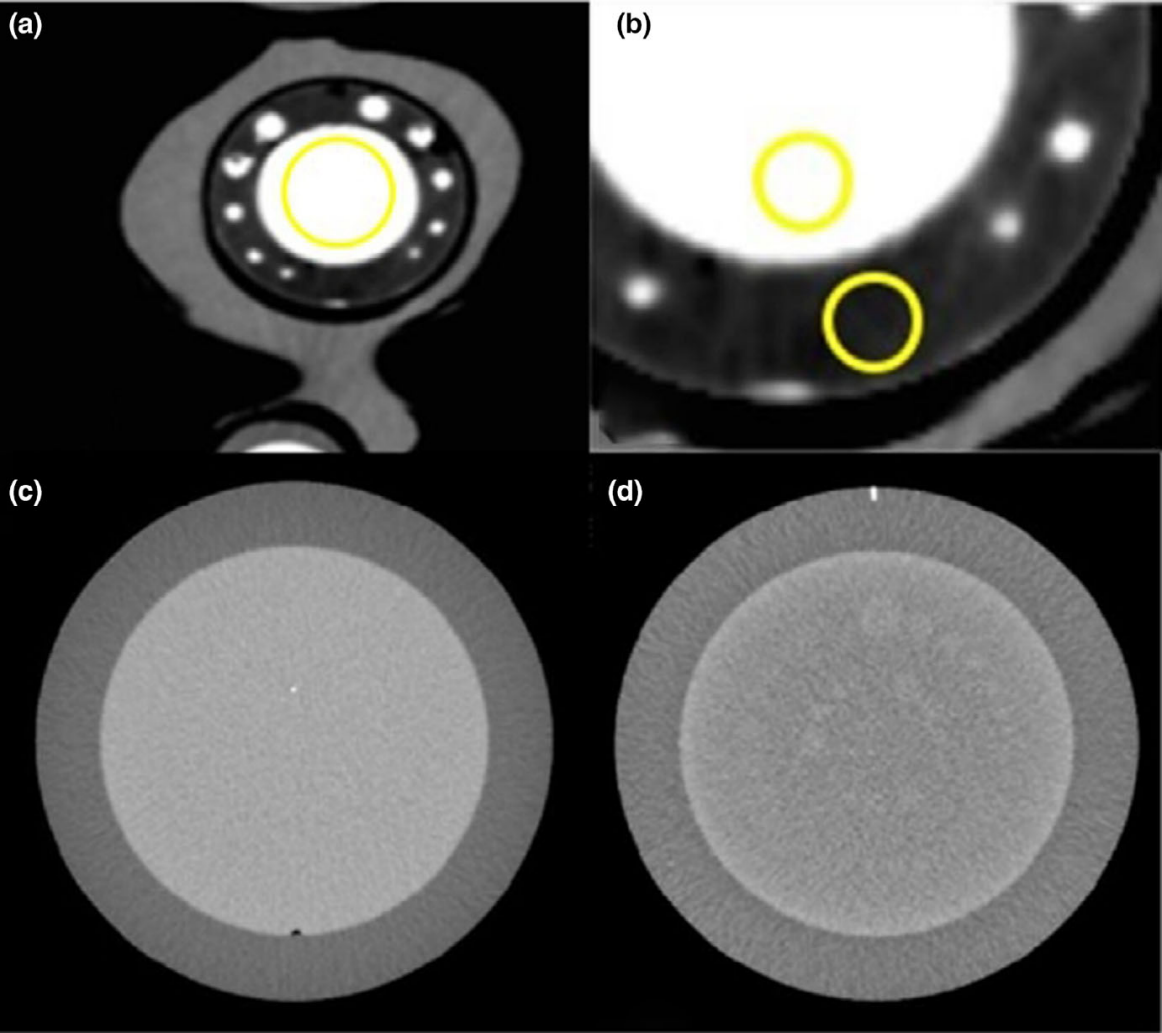
(a) To measure noise, an ROI was placed within the contrast‐enhanced region of the three‐dimensional (3D)‐printed cardiac insert phantom simulating the ascending aorta. (b) To measure CNR, two similar sizes of ROIs were placed between the contrast material (the ascending aorta) and the oil (fat). (c) For the evaluation of MTF (axial spatial resolution), the CTP528 module of Catphan^®^ 500 phantom was used. (d) For the evaluation of low contrast resolution, the CTP515 module of Catphan^®^ 500 phantom was used.

The attenuation values (Hounsfield Units, HU) and the image noise were measured by placing a region of interest (ROI) within the contrast‐enhanced region simulating the ascending aorta in the 3D‐printed cardiac insert phantom [Fig. 2(a)]. The signal–noise ratio (SNR) and the contrast–noise ratio (CNR) were calculated according to Eqs. (1) and (2), respectively. The CNR was obtained by using the HU values and image noise of the contrast material (CM) and the oil to simulate the myocardial fat in Eq. (2) [Fig. 2(b)].

(1) $$\text{SNR}=\frac{\text{HUcm}}{\sigma \text{cm}}$$

(2) $$\text{CNR}=\frac{\text{HUcm}‐\text{HUoil}}{(\sigma \text{cm}+\sigma \text{oil})/2}$$

The axial spatial resolution was measured using the images obtained from the CTP528 module of Catphan^®^ 500 phantom. First, the point spread function (PSF) was calculated from the scan of a small tungsten carbide bead (approximately 250 microns in diameter) [Fig. 2(c)]. Next, the line spread functions (LSF) were determined by integrating the PSF along vertical and horizontal directions. Last, the modulation transfer function (MTF) was calculated from the discrete Fourier Transform of the LSF datasets. The MTF values were automatically calculated by the AutoQA Lite^TM^ program with the output measures of MTF_50%_, MTF_10%_, and MTF_2%_.

The low contrast resolution was evaluated using the CTP515 module [Fig. 2(d)]. This module contains low contrast subslice targets with diameters of 2–15 mm, and contrast levels of 0.3%, 0.5%, and 1.0%. Low contrast resolution is the ability to differentiate objects with slightly different densities from background. The results of low contrast resolution were also generated and reported using the AutoQA LiteTM program.

Data analyses were carried out using Statistical Package for the Social Science (SPSS, version 21; IBM Corp., New York, NY, USA). Descriptive statistics, for example, mean and standard deviation, were calculated. Image noise, SNR, and CNR values were tested for normality by the Shapiro–Wilk test; thus, an ANOVA was used to examine the differences between image noise, SNR, and CNR.

## 3 RESULTS

### 3.A. Image noise, SNR, CNR, and contrast resolution

**Fig. 3:**
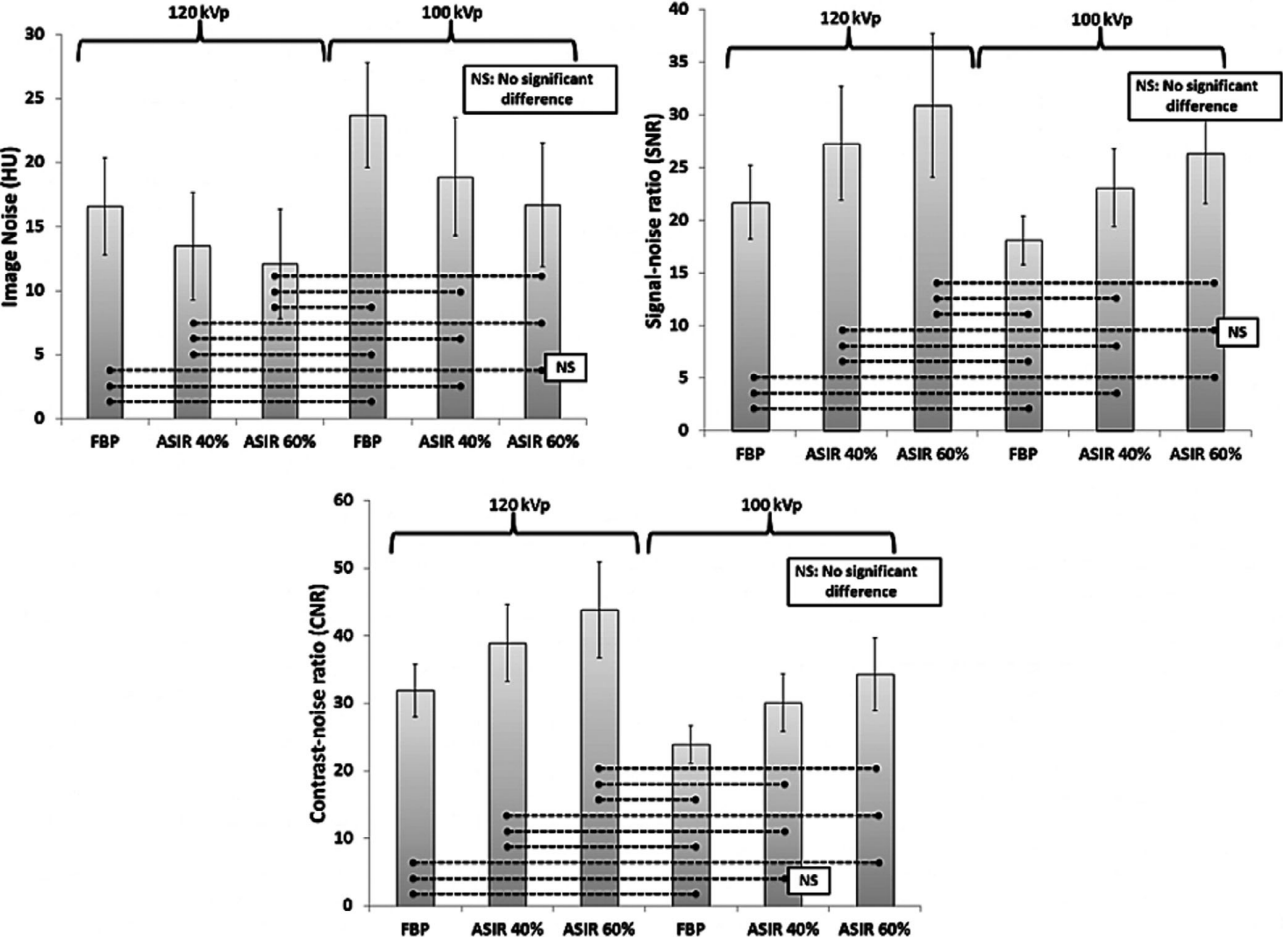
Bar graphs demonstrate significant differences in image noise (a), SNR (b), and CNR (c) (all *P* < 0.05) between the 120 and the 100 kVp.

There were significant differences between the measures of image noise, SNR, and CNR (all *P* < 0.001) for IR strengths and the tube voltage, but not for HU (Table 2). The simple main effect analysis showed that SNR and CNR of the image datasets of ASIR 40% and ASIR 60% were significantly higher than FBP, regardless of the tube voltage. The image noise was significantly lower and the SNR and CNR were significantly higher for the image datasets of 120 kVp tube voltage as compared to the 100 kVp (*P* < 0.001) (Fig. 3). The image datasets of ASIR 60% with 120 kVp resulted in the lowest image noise and the highest SNR and CNR (*P* < 0.05), whereas the FBP with 100 kVp protocol showed the highest image noise and the lowest SNR and CNR (Fig. 3). There was no significant difference in HU values between the image datasets of the 120 and 100 kVp (Fig. 3). There was no significant difference (*P* > 0.05) between 120 and 100 kVp measures of image noise of FBP vs ASIR 60% (16.6 ± 3.8 vs 16.7 ± 4.8), SNR of ASIR 40% vs ASIR 60% (27.3 ± 5.4 vs 26.4 ± 4.8), and CNR of FBP vs ASIR 40% (31.3 ± 3.9 vs 30.1 ± 4.3), respectively (Fig. 3).

**Table 2:** Results of HU, image noise, SNR, and CNR of the three‐dimensional (3D)‐printed cardiac insert phantom between 120 and 100 kVp.

Parameter	120 kVp					100 kVp			
	FBP	ASIR	ASIR	*P* value		FBP	ASIR	ASIR	*P* value
		40%	60%				40%	60%	
	Mean ± SD	Mean ± SD	Mean ±			Mean ±	Mean ±	Mean ±	
			SD			SD	SD	SD	
CT number (HU)	407.9 ± 6.4	408.0 ± 6.5	408.0 ± 6.5	NS		421.0 ± 5.6	421.0 ± 5.6	421.0 ± 5.6	NS
Image noise	15.0 ± 0.5	11.8 ± 0.5	10.4 ± 0.6		<0.001	22.2 ± 0.9	17.2 ± 0.8	15.0 ± 0.9	<0.001
Signal–noise ratio	23.2 ± 0.9	29.5 ± 1.6	33.7 ± 2.3		<0.001	19.0 ± 0.8	24.5 ± 1.3	28.2 ± 1.8	<0.001
Contrast–noise ratio	32.2 ± 2.3	40.4 ± 3.0	45.7 ± 3.8		<0.001	24.4 ± 2.4	31.1 ± 3.1	35.5 ± 3.7	<0.001

FBP, filtered back projection; HU, Hounsfield unit; IR, iterative reconstruction; SD, standard deviation.

Table 3 shows the MTF results obtained using the Catphan^®^ 500 phantom when the strengths of IR algorithm were increased. For both tube voltages, the spatial frequency of MTF was mildly affected by the different IR strength levels (variation < 10%). However, between 120 and 100 kVp protocols, the spatial frequency of MTF was strongly affected (variation > 10%), indicating changes in the spatial resolution and thus, image quality.

**Table 3:** The results of modulation transfer function (MTF) for FBP and different IR strength levels at 120 and 100 kVp.

Tube voltage (kVp)	Reconstruction settings	MTF 50% (lp mm^−1^)	MTF 10% (lp mm^−1^)	MTF 2% (lp mm^−1^)
120	FBP	0.416	0.659	0.806
	ASIR 40%	0.411	0.699	0.850
	ASIR 60%	0.414	0.720	0.888
100	FBP	0.370	0.702	0.804
	ASIR 40%	0.307	0.693	0.817
	ASIR 60%	0.297	0.686	0.840

ASIR, adaptive statistical iterative reconstruction; FBP, filtered back projection.

**Fig. 4:**
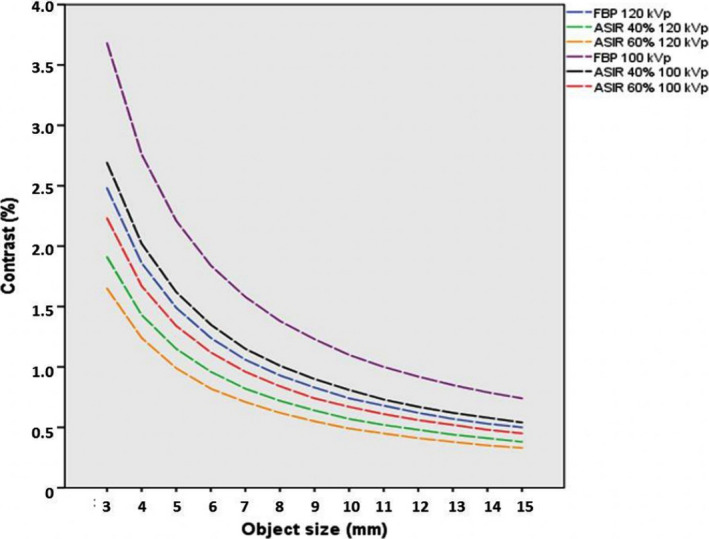
Results of low contrast resolution using CTP515 module of Catphan^®^ 500.

Figure 4 illustrates the changes in low contrast resolution for FBP and different strengths of IR at 120 and 100 kVp. The low contrast object diameter ranged from 3 to 15 mm. From the graph, the image datasets reconstructed with FBP at 100 kVp required the highest contrast to detect the smallest size of object diameter of 3 mm. The ASIR 60% at 100 kVp has a higher contrast resolution compared to the FBP at 120 kVp for the smallest object diameter. The highest contrast resolution was the ASIR 60% at 120 kVp.

## 4 DISCUSSION

This study used a 3D‐printed cardiac insert and Catphan^®^ 500 phantoms to investigate the effect of varying IR strengths at different tube voltages for CCTA acquisition protocols. The results demonstrated that the IR ASIR 60% with a low tube voltage of 100 kVp produced comparable SNR to the current local CCTA protocols of 120 kVp and ASIR 40% resulting in a > 50% reduction in radiation dose as measured by the CTDI_vol_. In our previous systematic review,^22^ we concluded that using an IR algorithm allows up to 41% dose reduction as compared to FBP while maintaining similar image quality. Our findings have revealed that although the IR algorithm is already a powerful tool to reduce dose, the higher IR strength at a lower tube voltage has resulted in a further dose reduction.

Our study adds to existing data by investigating the combination of higher IR algorithms strengths and low tube voltage at CCTA using two different phantoms, 3D‐printed cardiac insert and Catphan^®^ 500 phantoms. These phantoms allow us to adequately evaluate image quality characteristics such as image noise, SNR, CNR, spatial resolution, and low contrast resolution. Consequently, the 3D‐printed cardiac phantom in conjunction with Catphan^®^ 500 phantom can be used as a tool for dose optimization in clinical CCTA studies.

Several limitations of our study merit consideration. First, this investigation was conducted at a single center using a 64‐slice CT scanner, and thus, the results may not be applicable to other institutions where different types of CT scanners (e.g., 128 or 320‐slice), protocols, and IR algorithms settings are used. Second, all scans were performed on the phantoms without cardiac motion. Cardiac motion is an important factor in CCTA examination that could produce artifacts and deteriorate the image quality. Therefore, we are planning to develop a heart‐beating cardiac insert phantom in the future that may better simulate CCTA images and also performing this investigation at multicenter levels. Third, comparison of image quality measurements to the patient image datasets would improve the current results. Fourth, visual image quality was not assessed by human observers limiting the findings to quantitative measures of image quality.

## 5 CONCLUSION

Changing the IR strength has yielded different image quality noise characteristics. In this study, the use of 100 kVp and ASIR 60% yielded comparable image quality noise characteristics to the standard CCTA protocols using 120 kVp of ASIR 40%. In addition, a combination of 3D‐printed and Catphan^®^ 500 phantoms could be used to perform CT dose optimization protocols.

## CONFLICT OF INTEREST

No conflict of interest.
